# P32 A pragmatic quality improvement ‘good enough’ approach to measuring and comparing carbon-footprint savings associated with ‘Go Green’ switching from IV to oral therapy (IVOST) or reducing IV dose frequency

**DOI:** 10.1093/jacamr/dlaf118.039

**Published:** 2025-07-14

**Authors:** Rachael Rodger, Rosie Hillson

**Affiliations:** NHS Greater Glasgow and Clyde, Glasgow, UK; Centre for Sustainable Healthcare, UK

## Abstract

**Background:**

Antimicrobial stewardship (AS) quality improvement (QI) initiatives promoting IV to oral switch (IVOST)^1^ or reduced IV dosing frequency provide benefits for patients, staff and the environment.^2,3^ To determine if initiatives are effective, we need to measure if improvement has occurred. Measuring carbon-footprint savings can be challenging due to lack of available information on the environmental impact of pharmaceutical products and the time needed to complete a thorough environment impact calculation. Consequently, more pragmatic ‘good enough’ QI indicator measures need to be developed to enable improvement in the environmental impact of QI initiatives promoting reduced IV use to be more easily measured and compared.^4^ One such approach would be to remove drug and reconstitution products from the calculation^5^ and focus on the equipment commonalities used in the standard process of peripheral IV administration. This approach enables standardized indicator measures for carbon-footprint savings in terms of plastic waste reduction to be developed.

**Objectives:**

To measure the carbon-footprint saving associated with switching from peripheral IV to solid oral administration in terms of plastic waste reduction and use this to develop standard indicator measures for calculating carbon-footprint savings associated with IVOST or reduced IV dosing frequency.

**Methods:**

Using process mapping the core administration equipment for common peripheral IV dosing schedules was collated over a 72 h period in line with local policy for replacement of peripheral lines. A hybrid carbon foot-printing methodology was used to estimate the carbon-footprint of peripheral IV and oral medicine administration in terms of carbon dioxide equivalent (CO_2_e) emissions.^6,7^ Emissions associated with the IV and oral pharmaceutical products were excluded. A process-based approach was used to estimate emissions of the syringe, cannula, safety needle and oral medicine plastic pot using emission factors taken from the UK government carbon conversion factors database^6^. In addition, an environmentally extended input output analysis (EEIOA) was used to estimate the emissions associated with alcohol wipes, saline solution, IV3000, Octopus 2 and Volumat Line using the medical instrument conversion factor taken from the 20/21 Greener NHS database. Emissions associated with non-sterile gloves and aprons were taken from a previously published report^7^.

**Results:**

This pragmatic approach enabled CO_2_e savings in terms of plastic waste reduction for IVOST of common IV dosing schedules to be calculated irrespective of the drug administered (Table 1). This methodology also enabled CO_2_e savings to be calculated and compared between different drugs when switching from IV to oral administration at the same/different dosing frequency or when switching from higher frequency (e.g. 4-6 times daily) to lower frequency (once or twice daily) IV regimens (Tables 1 and 2) or to extended interval IV regimens given weekly or monthly.

**Conclusions:**

Taking a pragmatic ‘good enough’ approach to measuring improvement in carbon-footprint savings associated with IVOST enabled standard indicator CO_2_e savings in terms of plastic waste reduction to be calculated. This is important in supporting more efficient, straightforward and comparable measurement of carbon-footprint savings from AS QI initiatives promoting IVOST or switching from higher to lower frequency dosing schedules of different drugs.

**Table 1.** Comparison of the carbon footprint of common peripheral IV dosing schedules and savings of IVOST in terms of plastic waste reduction

Administration schedule	IV KgCO_2_e per dose	Oral KgCO_2_e per dose	KgC02e saving per IVOST dose	KgCO_2_e saving per IVOST day
Once daily dosing	1.6235	0.0067	1.6168	1.6168
Twice daily dosing	1.4156	0.0067	1.4089	2.8178
Three times daily dosing	1.3468	0.0067	1.3401	4.0203
Four times daily dosing	1.3122	0.0067	1.3055	5.2220
Six times daily dosing	1.2777	0.0067	1.2710	7.6260

Calculations exclude drug and reconstitution products.

**Table 2.** Examples of comparison of the carbon footprint savings in terms of plastic waste reduction of peripheral IVOST of different dosing frequency regimens or reducing IV dosing frequency

Change in administration regimen	KgCO_2_e saving per day
**Six times daily IV flucloxacillin** switched to **ONCE daily IV daptomycin**	**6.0427** (1.2777 × 6) − 1.6235
**Twice daily IV vancomycin** switched to **BD oral linezolid**	**2.8178** (1.4156 × 2) − (0.0067 × 2)
**Four times daily piperacillin/tazobactam** switched to **TDS oral co-amoxiclav**	**5.2287** (1.3122 × 4) − (0.0067 × 3)
**Three times daily IV meropenem** switched to **ONCE daily IV ertapenem**	**2.4169** (1.3468 × 3) − (1.6235)

Calculations exclude drug and reconstitution products.
